# Diseases of Digestive Organs

**Published:** 1906-05-12

**Authors:** 


### DISEASES OF DIGESTIVE ORGANS.

**Physiology of Digestion.**—In an exhaustive and clearly expressed paper Stirling [1] gives an account of the recent advances in the subject. Physiology is becoming every year more important as a basis of practical medicine, and the recent work of Pawlow and Starling has already had its effect on the pathology and treatment of certain disorders. Beginning at the mouth, the function of the saliva is to act as a solvent and lubricant, as well as to convert some starchy material into maltose. The solvent action enables the sense of taste to be exercised, which with other senses (olfactory, thermal, etc.) is of importance in making the animal immediately aware of the *kind* of substance which is taken into the body. If it is unacceptable nausea is reflexly set up, and the substance is expelled; at the same time a copious secretion of watery saliva takes place, washing out thoroughly the buccal cavity. Dry food excites a watery secretion from the parotid; soft, wet food causes no such effect. The whole mechanism is under the direct control of the nervous system, and is a reflex one, excited (1) psychically, by the thought or sight of food; (2) by taste; (3) by direct contact apart from taste, as is seen when tasteless substances are taken into the mouth. The reaction must needs be rapid, hence it is nervous, not chemical, in nature. In the stomach Pawlow has shown that the psychic element is most important in producing the free secretion of active gastric juice, hence the importance to digestion of a good appetite and an unclouded mind. In dogs fed without their knowledge through a gastric fistula, digestion was much less active than in those in whom in addition "sham feeding" by the mouth was also practised. "Sham feeding" consists of introducing food into the mouth in a dog in whom the œsophagus has been divided, so that it merely conducts its contents out at a fistula in the neck, and not into the stomach. The secreto-motor nerves to the stomach are the vagi. Certain substances have a direct stimulating action on the gastric secretion, apart from psychic influence; these are meat juice, meat extracts, milk and gelatine solutions, peptones, dextrin, etc. The value of such substances as foods is therefore quite separate from their actual nutritive constituents. But besides this nervous secreto-motor effect, there is another mechanism in gastric digestion—namely, the chemical reflex demonstrated by Edkins. The pyloric mucous membrane secretes a substance under the stimulus of acid food called gastric secretin or gastrin, which is absorbed into the bloodstream and carried to the glands of the fundus, exciting a second and less abundant secretion of gastric juice. The pyloric sphincter is from time to time opened to allow of the passage of acid chyme into the duodenum. As long as the contents of the first part of the duodenum are acid the pylorus is reflexly stimulated, and remains contracted; as soon as they become alkaline the stimulus is removed, and the pylorus relaxes, allowing a further escape of chyme from the stomach. With the beginning of the duodenum the influence of the nervous system wanes, and in its place a system of chemical reflex action begins—slower and more continuous in action. The duodenal mucosa contains a substance pro-secretin, which is activated by the acid chyme, and becomes secretin. This, absorbed and carried to the pancreas, liver and intestinal walls, stimulates their activity and produces a copious formation of thin secretions. The effect of bile succus entericus is mainly adjuvant to that of the pancreatic juice. Secretin is not a ferment, but belongs to the same class of bodies as adrenalin. The pancreatic juice varies according to the kind of food habitually taken; hence digestion is likely to be disturbed by the ingestion of unwonted food-stuffs. It has even been shown that lactose, if ingested, calls forth a special secretin in the mucosa of the duodenum, which stimulates the pancreas to produce the appropriate enzyme lactase for its digestion. In the intestine, secretion is most active in the upper part, and decreases to nil downwards, while absorption is slight in the duodenum and becomes progressively more active downwards. The succus entericus, besides the secretins, contains several ferments. One, enterokinase, is the ferment of ferments which specially activates the proteolytic ferment of the pancreas, invertase, which alters cane sugar into a monosaccharid, and maltase which forms glucose from maltose. It also contains erepsin, which splits up the products of pancreatic digestion of albumin into still simpler compounds.

**Xerostoma.**—A curious instance of the effect of the nervous system on the secretions of the upper part of the alimentary tract is seen in this somewhat rare condition, an instance of which has recently been reported by Sowers.[2] Xerostoma, or dry mouth, which in relatively slight degrees is not very uncommon especially in the aged, may occur to an excessive degree in certain persons usually concomitantly with some nervous shock, or the appearance of marked neurotic tendencies. It usually is seen in elderly people. As a result of the total or almost total suppression of the buccal and salivary secretions, the mucous membrane of the mouth, pharynx, larynx, etc., becomes dry and glazed. Mastication and deglution are much impeded, and great discomfort is experienced. Epithelial and food débris collects in hard dry heaps on the mucosa, and often there is constant spitting of plugs of viscid semi-solid matter, the only remaining product of the salivary and mucous glands. The teeth often fall out or become carious, taste is impaired or lost, weight decreases, and there is often pain and swelling of the salivary and lymphatic glands, probably owing to an ascending infection from the mouth. Somers, in his review of the published cases, shows that drugs have practically no influence on the disease, but seems to recommend peroxide of hydrogen as an efficient cleanser of the mouth.

**Examination of the Stomach Contents.**—It is obvious that without a thorough examination of the fundamental activity of the stomach both as regards its secretory and motor power, no diagnosis of the severer cases of digestive disturbance can efficiently be made. Hewes,[3] in order to determine the motor power of the stomach, withdraws the stomach contents twelve hours after a test meal and examines it chemically and microscopically. If there is stasis, *i.e.*, a mechanical impediment to the passage of food into the duodenum, one or more of the following conditions will be observed: (1) Food residues; (2) sarcinæ; (3) lactic acid; (4) abnormal yeast fermentation. Sarcinæ generally occur when free HCl is present, yeast fermentation is also only possible when free HCl occurs. It is measured by mixing 2 c.c. of gastric contents with 10 c.c. of 10 per cent. glucose solution and measuring the gas given off in 24 or 48 hours. More than 1 c.c. of gas is abnormal and freshly budding yeast cells will be found in the deposit. Bacterial fermentation goes with the presence of lactic and absence of hydrochloric acid. Occult bleeding should also be tested for, as in its absence active ulcer, carcinoma, or stenosing gastritis may be excluded. The gastric contents are treated with one-third glacial acetic acid extracted with ether and tested with guaiacum. The determination of the acids is of less value than the detection of stasis; but if the latter is present, absence of free HCl and presence of lactic acid is in favour of cancer as against ulcer. If the size and position of the organ are normal, stasis is due to organic stenosis, if much dilated or ptosed, the diagnosis is not so certain. When the use of the stomach tube is not possible, Sahli's desmoid reaction[4] may be tried. After an ordinary meal a rubber capsule containing iodoform and methylene blue and tied up with ordinary catgut is swallowed. The stomach-juice can dissolve the catgut, but not so the pancreatic secretion. The urine is tested for the presence of iodine and methylene blue six hours after the meal. Occasionally this test is fallacious, but it is often valuable, especially if no HCl is found after a test breakfast, a negative reaction then denotes the presence of serious organic disease.

[1] Med. Chron., Dec., 1905, p. 141. [2] Ann. Jour. Med. Sci., Sept., 1905, p. 451. [3] Edinburgh Med. Jour., Feb., 1906, p. 168. [4] Ibid.

